# Adaptive Modeling of Tandem Mass Spectrometry Data: Creation of the METLIN 960K MRM Database

**DOI:** 10.1021/acs.analchem.5c04639

**Published:** 2025-10-28

**Authors:** Aries Aisporna, Bill Webb, Winnie Uritboonthai, Linh Hoang, Elizabeth M. Billings, Corey Hoang, Robert Plumb, Gary Siuzdak

**Affiliations:** † Scripps Center for Metabolomics and Mass Spectrometry, 4356 The Scripps Research Institute, 10550 North Torrey Pines Road, La Jolla, California 92037, United States; ‡ Centre for Metabolomics Research, University of Liverpool, Liverpool L69 7ZB, U.K.; § Department of Integrative Structural and Computational Biology, Department of Chemistry, 4356 The Scripps Research Institute, La Jolla, California 92037, United States

## Abstract

Multiple Reaction Monitoring (MRM) remains the gold standard for quantitative mass spectrometry but continues to be constrained by the limited availability of high-quality transitions and collision energy (CE) values for many biologically and chemically relevant molecules. Here, we present the METLIN 960K MRM library, a 960,000-compound transition resource derived entirely from empirically acquired MS/MS data. MRM transitions were generated in both positive and negative ionization modes using an empirical spline-based pipeline refined by AI BioSync, an XCMS enhancement that provides a framework of AI and machine-learning tools designed to decipher spectral data for biological and analytical relevance. Central to this approach is spline fitting of CE-dependent intensity profiles from experimental MS/MS data collected at four discrete energies (0, 10, 20, and 40 eV), enabling continuous CE modeling and precise prediction of optimal fragmentation conditions. Supervised learning models were used within AI BioSync to refine spline fitting across diverse chemical classes, improving reproducibility and predictive accuracy. Validation across more than 100 authentic compounds, including rare metabolites and diverse small molecules, demonstrated robust detection down to 1 nM, confirming both sensitivity and scalability. This framework also holds immediate applicability for preclinical drug development studies, where authentic metabolite and impurity standards are often unavailable. Unlike prior methods reliant on *in silico* fragmentation or heuristic rules, all transitions are derived directly from experimental MS/MS data using absolute intensities. The resulting precursor *m*/*z*–centric METLIN 960K MRM library (https://metlin.scripps.edu) greatly expands the chemical space accessible to targeted quantitation, providing a scalable, vendor-independent path for sensitive and specific molecular detection across research, clinical, and applied applications.

## Graphical abstract

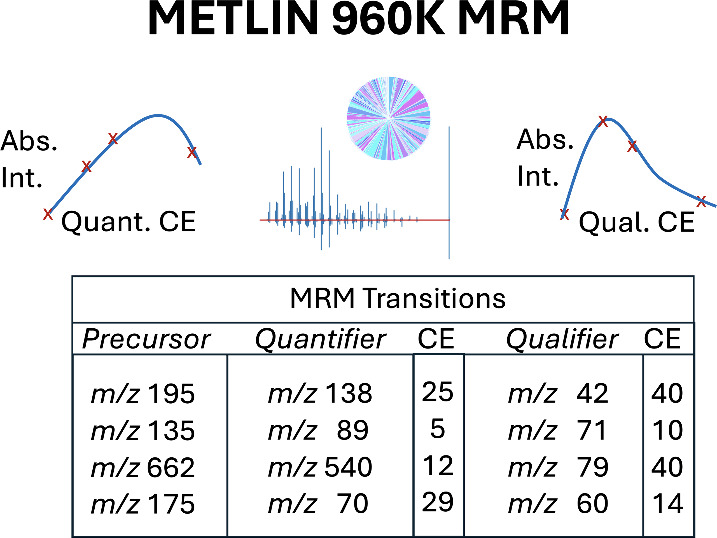

## Introduction

Multiple reaction monitoring (MRM) mass spectrometry remains the gold standard for targeted quantitative analysis owing to its unparalleled sensitivity, specificity, speed, and reproducibility.^1–10^ However, development of high-quality MRM transitions typically depends on access to pure standards and manual optimization of fragment ions and collision energies (CEs), making the process time-consuming and limited in scale. Prior community-based resources, such as METLIN-MRM and XCMS-MRM,^10^ provided more than 15,000 transitions by combining experimental data with heuristic rules—important early steps toward democratizing MRM design.^11–14^ Yet these approaches were restricted by static CE assumptions, relative-intensity data, and limited molecular coverage.

In parallel, the broader landscape of MRM resources comprises a mix of commercial, community, and vendor-specific tools that facilitate transition selection and method development. Skyline (open-source) is the most widely adopted platform, enabling users to build and share MRM assays from experimental or vendor-supplied data; however, its accuracy ultimately depends on user-defined input and the limited size of publicly available small-molecule transition libraries. MRMPilot (SCIEX) and Compound Discoverer (Thermo Fisher) provide vendor-integrated solutions for CE optimization and scheduled MRM design but are confined to predefined compound lists and specific instrument families. Community and vendor repositories such as SRM Atlas and MRMaid offer curated transitions yet focus primarily on peptides, with small-molecule coverage generally limited to hundreds or a few thousand analytes. Additional vendor resources, including Agilent PCDL and Waters Quanpedia, contain high-quality transitions but remain proprietary and instrument-specific, limiting interoperability.

In contrast, METLIN 960K MRM uniquely scales this concept to nearly one million empirically acquired small-molecule spectra encompassing metabolites, lipids, drugs, natural products, and environmental compounds. Each entry includes positive- and negative-mode MS/MS data collected at four discrete collision energies (0, 10, 20, 40 eV), enabling comprehensive modeling of CE-dependent fragmentation.

Here, we introduce an empirical spline-based framework, refined through AI-guided optimization, that expands MRM transition prediction to over 960,000 small molecules using METLIN’s high-resolution MS/MS library.^15,16^ By modeling fragment-ion behavior across four collision energies with spline fitting, the system captures CE-dependent intensity dynamics to predict optimal quantifier and qualifier ions together with their collision energies. AI served as a refinement layer to evaluate and optimize spline-fitting performance across chemical classes, improving accuracy and reproducibility. This data-driven strategy avoids traditional rule-based shortcuts, instead selecting transitions based on intensity, CE stability, and *m*/*z* distance from the precursor, thereby enhancing robustness and analytical performance.

The resulting METLIN 960K MRM library enables transition generation even in the absence of commercial standards, offering broad coverage across diverse compound classes in both ionization modes. It provides a scalable platform for high-throughput, reproducible quantitation and lays the groundwork for standardized targeted analysis, with accuracy validated against experimental data and supported by ongoing large-scale benchmarking efforts.

## Methods

### Data Source and Spectral Preprocessing

All MS/MS data were sourced from the METLIN tandem mass spectral database, comprising over 960,000 empirically acquired and authenticated chemical standards.^10,15,16^ These standards represent a broad diversity of molecules, including metabolites, lipids, drugs, natural products, toxicants, environmental contaminants, and synthetic intermediates. Compounds were acquired from individual laboratories, academic consortia, government agencies, regulatory bodies, biotechnology and chemical companies, research institutes, instrument vendors, environmental and agricultural testing laboratories, clinical and forensic laboratories, pharmaceutical companies, and nonprofit foundations.

High-resolution MS/MS spectra were collected using both direct infusion and LC–MS/MS methods in both positive and negative ionization modes across four standardized collision energies (0, 10, 20, and 40 eV), primarily on Agilent quadrupole time-of-flight (QTOF) mass spectrometers, with additional data contributed from Thermo Orbitrap, Bruker QTOF, and SCIEX QTOF instruments. Only MS/MS spectra derived directly from authenticated reference standards were included in METLIN; no in silico–predicted or library-propagated spectra were used. Raw spectra were archived as vendor-format files and converted to centroided data (.mzML) for processing using ProteoWizard v3.0.24115.

### Fragment Tracking and CE Profiling

Fragment ions were dynamically tracked across the four collision energies (0, 10, 20, and 40 eV) using a ± 0.5 Da mass tolerance to group recurring fragment ions corresponding to the same molecular feature. For each compound, all MS/MS spectra were first centroided and fragment alignment across collision energies was performed using an iterative *m*/*z* matching algorithm that prioritized consistent signal detection across at least three of the four CEs.

Each matched ion group was assigned a collision energy–intensity profile, capturing the empirical change in fragment abundance as a function of CE. This CE-dependent behavior was subsequently used to construct continuous fragmentation trends through spline fitting (see *Collision Energy Prediction*). To ensure accuracy, fragments appearing in only a single CE or exhibiting nonmonotonic or discontinuous behavior were excluded from downstream modeling to minimize false associations from in-source fragments or isotopic interferences.

Quantifier and qualifier transitions were selected based on the reproducibility and signal consistency of each fragment across the CE range. The quantifier ion was defined as the most intense and stable fragment within the CE–intensity profile, while the qualifier ion was typically the next-highest intensity fragment meeting reproducibility and mass separation criteria (≥2.0 Da from the precursor). This approach ensured that selected transitions reflected reproducible, energy-dependent fragmentation behavior rather than single-point or instrument-specific noise.

### Collision Energy Prediction and AI Refinement

Collision energy (CE) predictions were generated using univariate spline fitting (cubic splines; *k* = 2, smoothing factor *s* = 0) for each fragment’s CE–intensity curve. The predicted optimal CE corresponded to the spline curve maximum. Values below 5 eV or outside the tested range were clamped to 5 eV, reflecting the lower limit for standard triple-quadrupole operation.

While spline fitting forms the core of the prediction framework, AI-guided optimization implemented within an AI BioSync environment was introduced to improve generalization and reproducibility across chemical classes. Specifically, supervised regression models implemented in Python v3.11 using scikit-learn v1.4.0 (*RandomForestRegressor* and *GradientBoostingRegressor*) evaluated the relationship between empirical and predicted CEs to identify the most robust spline-fitting conditions. Several large-language-model (LLM) systems, ChatGPT 5, Grok, and Gemini, were used interactively to assist in conceptual algorithm development, code refinement, and comparative testing of regression strategies. All final code and analyses were performed by the authors, with AI tools serving only as aids to improve computational efficiency and model selection.

Spline calculations and CE–intensity visualizations were performed in R v4.3.2 using the *splines*, *mgcv*, and *ggplot2* libraries. Large-scale MRM generation was executed on the Garibaldi 64-bit Linux computing cluster (2,848 cores; 250 TB DDN SFA10K storage with IBM GPFS; Torque queue system).

### Transition Selection Criteria

For each compound, one quantifier and one qualifier transition were selected. Fragment ions were ranked by relative intensity at their predicted optimal CE. To ensure compatibility with standard MRM configurations, only fragments at least 2.0 Da lower than the precursor *m*/*z* (±0.1 Da) were considered. The >2 Da threshold was adopted empirically after observing that smaller Δ*m*/*z* transitions frequently reflected precursor-related artifacts, while no valid transitions occurred beyond that cutoff. Users requiring stricter selectivity (e.g., > 7 Da separation) can filter transitions accordingly from the provided data sets.

All selected transitions were output as [precursor *m*/*z*] → [product *m*/*z*] pairs with associated CE values for integration into instrument acquisition methods. To streamline algorithm development and improve reproducibility, no explicit adduct designations were included. Introducing adduct size and type as an additional variable (e.g., [M + H]^+^, M^+^, [M + Na]^+^, [M+NH_4_]^+^, [M–H]^−^, etc.) made it difficult to programmatically identify the true precursor and accurately map fragment relationships across large data sets. By designating the measured precursor as the central reference point, a precursor-centric approach, we removed this variable and focused solely on optimizing transition generation. This simplification improved accuracy, algorithmic stability, and practical usability across diverse compound classes and ionization modes. Adduct-specific details can still be inferred from molecular formula and precursor *m*/*z* information provided within METLIN.

### Benchmarking and Validation

METLIN-predicted transitions were validated against a benchmark set of experimentally optimized MRM transitions acquired on Agilent 6495*B*/6465B, Waters Xevo TQ-S/TQ-XS, Thermo TSQ Altis, and SCIEX API 3200 instruments. Evaluation metrics included:^1^ fragment *m*/*z* agreement within ± 0.5 Da,^2^ CE prediction error (ΔCE), and^3^ transition overlap with manually optimized quantifier and qualifier sets.

Validation included 30 molecules across biologically and clinically relevant classes and a mixture of >80 diverse standards^17^ analyzed at 1 μM and 1 nM concentrations to assess sensitivity. Combined, these analyses confirmed accurate, sensitive, and reproducible transition prediction across >100 compounds.

### Software and Availability

All algorithms for fragment tracking, spline-based CE prediction, and transition selection were implemented in Python using the Pandas, NumPy, and SciPy libraries. Jupyter Notebooks were used for pipeline development and visualization..

All algorithms for fragment tracking, spline fitting, CE prediction, and AI optimization were implemented in Python v3.11 (*pandas v2.2.2, numpy v1.26.4, scipy v1.13.1, scikit-learn v1.4.0*) and R v4.3.2 (*splines*, *mgcv*, *ggplot2*). Jupyter Notebooks were used for pipeline development and visualization. Code is available upon request, and the predicted transitions can be accessed via the METLIN web platform (https://metlin.scripps.edu).

## Results and Discussion

To address the persistent challenge of large-scale MRM transition generation,^10–17^ we developed an empirical strategy leveraging high-resolution MS/MS spectra acquired from authentic standards within the METLIN database. Using spectral data collected at four distinct collision energies (0, 10, 20, and 40 eV), spline fitting was applied to model absolute ion intensity behavior across the energy range. This framework enables the identification of both quantifier and qualifier ions for each molecule, based on absolute intensity and collision energy specificity.

AI was incorporated as a refinement layer rather than a replacement for spline modeling. Supervised learning models were used to evaluate alternative spline fits across diverse chemical classes, helping identify approaches that produced the most reproducible and accurate predictions. This integration minimized outliers and improved robustness without altering the fundamentally empirical, data-driven nature of the pipeline.

Rather than relying on theoretical predictions or in silico fragmentation, this method is grounded in experimentally derived spectra, with AI serving to enhance quality control and class-wide consistency. The resulting transitions therefore combine the reliability of empirical MS/MS data with the scalability afforded by automated modeling and refinement.

Figure 1 illustrates the foundational concept of this approach. The algorithm analyzes intensity traces for each fragment ion to determine optimal collision energies and rank ions by suitability as quantifier or qualifier transitions. The output is a table indexed by METLIN Identification Number (MID), linking each compound to its corresponding MRM transitions. This resource, built directly from over 960,000 authentic molecular standards, provides a scalable solution to assay development and represents a marked advancement over previous transition libraries that relied heavily on curated or predicted data. For consistency across the entire 960K-compound resource, no explicit adduct designations are assigned. Transitions are reported solely by measured precursor *m*/*z* and its corresponding fragment ions, ensuring that selection criteria remain uniform regardless of ionization chemistry.

**Figure 1:**
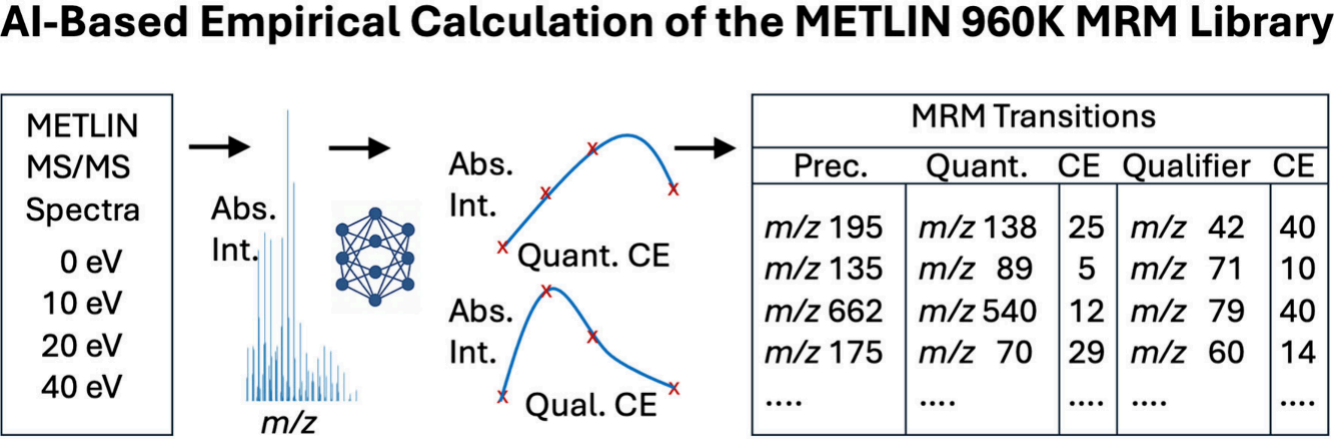
High-resolution MS/MS spectra acquired at four collision energies (0, 10, 20, and 40 eV) are used to train an AI generated algorithm that models intensity profiles across CEs. The system selects optimal quantifier and qualifier ions for each compound, along with their predicted collision energies. The resulting output is a transition table indexed by METLIN Identification Number (MID), enabling the generation of MRM assay development using data from the authentic standards within METLIN.

To operationalize this strategy at scale, we developed a streamlined pipeline (Figure 2) for empirical MRM transition assignment across METLIN’s entire MS/MS data set. The workflow begins by loading METLIN’s MS/MS spectra acquired at 0, 10, 20, and 40 eV, followed by tracking recurring fragment ions across these collision energies to evaluate their CE-dependent intensity profiles. This step enables the exclusion of low-intensity ions and nonspecific in-source fragments (ISF), while favoring fragments that exhibit reproducibility and CE-dependent stability.

**Figure 2:**
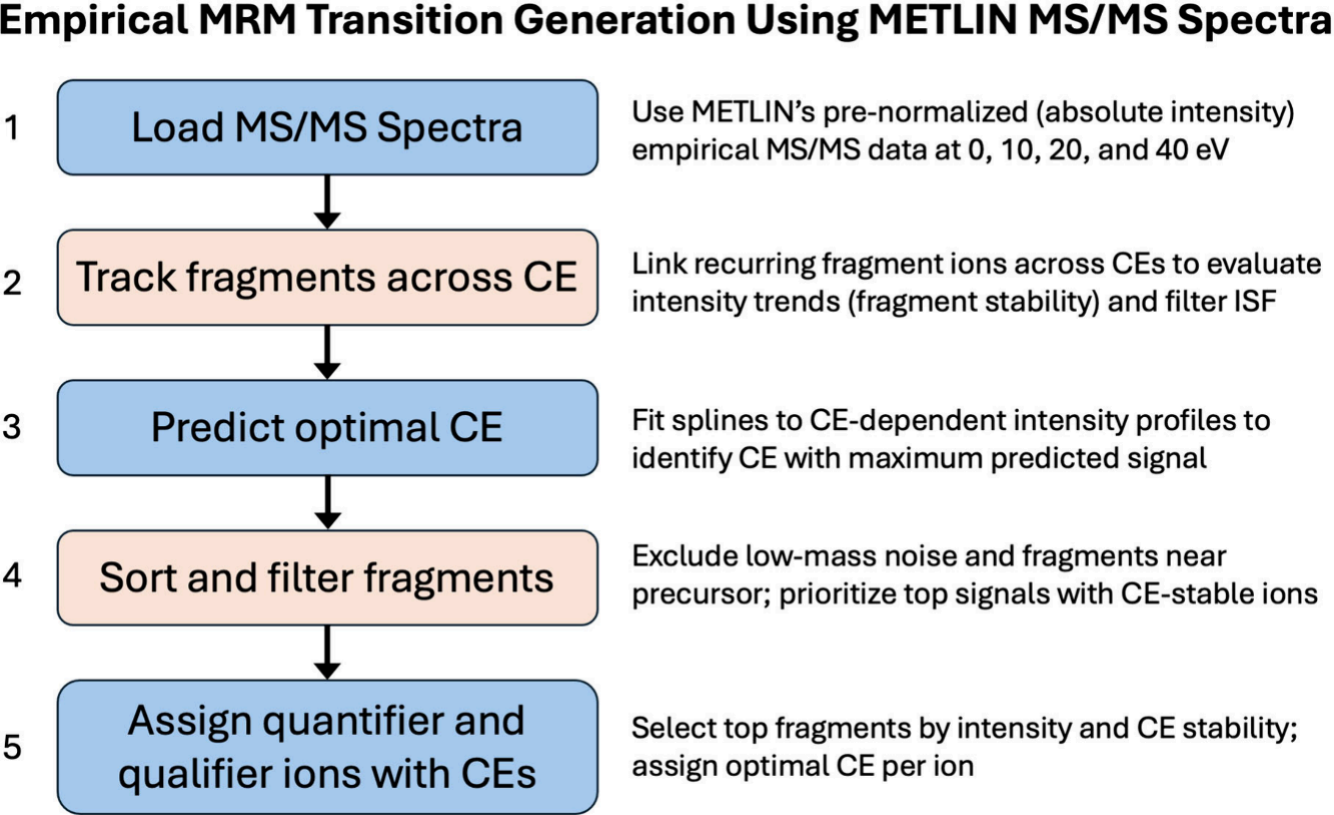
Workflow for METLIN-based calculation of MRM transitions. An empirical spline pipeline, refined by AI for class-wide consistency, processes METLIN’s MS/MS data across four CEs (0, 10, 20, and 40 eV) to generate high-confidence MRM transitions. Steps include: (1) loading MS/MS spectra, (2) tracking fragments across CE to evaluate stability and avoid nonspecific in-source fragments (ISF), (3) using spline fitting to predict the optimal CE per ion, (4) sorting and filtering fragments to exclude low-intensity or precursor-adjacent ions, and (5) assigning quantifier and qualifier ions with their corresponding optimized CEs.

Once the CE trajectories are established, spline fitting is applied to each ion’s intensity curve to identify the collision energy at which it produces maximal signal. Fragments are then ranked by both intensity and CE selectivity, with the top candidate assigned as the quantifier ion and secondary candidates selected as qualifiers. The result is a set of empirically grounded transitions and optimized collision energies for each compound, all indexed by METLIN ID. Importantly, this approach avoids reliance on heuristic rules or predictive fragmentation models, instead building the MRM library entirely from measured spectral behavior—dramatically expanding the coverage, accuracy, and reliability of MRM assays across chemical space.

To illustrate the behavior of the algorithm and the resulting fragment selection, we examined several representative molecules, and their fragmentation intensity profiles across collision energies (Figure 3). Glutamic acid, caffeine, and arginine were selected as examples due to their widespread relevance and well-characterized MS/MS behavior. For each molecule, the absolute intensity of key fragment ions was plotted across the four experimental CEs, and splines were fitted to estimate the optimal CE at which each fragment reaches maximal abundance.

**Figure 3:**
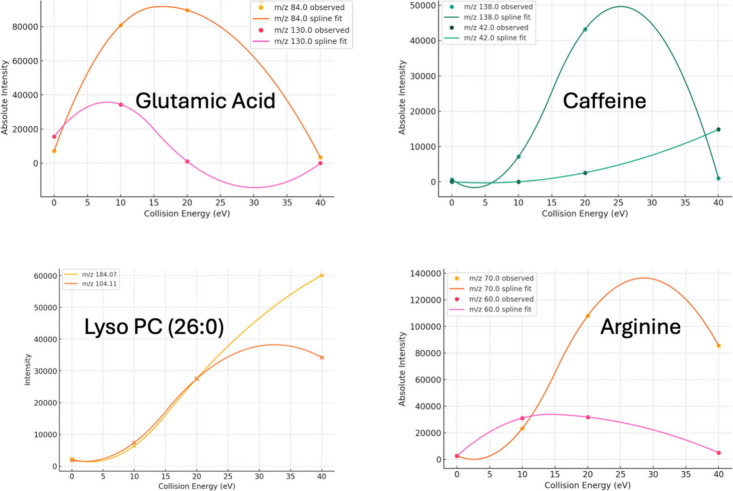
Collision energy-dependent fragmentation profiles (splines).Observed absolute intensities (points) and spline fits (lines) are shown for the quantifier and qualifier ions of each compound across four collision energies (0, 10, 20, and 40 eV) for each molecule (glutamic acid, lysoPC, arginine and caffeine). Optimal collision energies were determined from the absolute intensity profiles, providing empirical guidance for MRM transition selection.

This visualization highlights the rationale behind quantifier and qualifier selection. For example, in glutamic acid, the ion at *m*/*z* 84.0 shows a clear CE-dependent peak near 20 eV, while the qualifier ion at *m*/*z* 130.0 exhibits lower overall intensity and a less defined maximum—validating its role as a secondary confirmatory transition. Similar behavior is observed in arginine, where the quantifier (*m*/*z* 70.0) dominates at higher CE values compared to the lower-intensity qualifier (*m*/*z* 60.0). In caffeine, both the quantifier (*m*/*z* 138.0) and qualifier (*m*/*z* 42.0) demonstrate distinct CE profiles, peaking at different energies, thereby ensuring both signal strength and orthogonality in transition selection. These examples confirm the program’s ability to select meaningful and experimentally aligned MRM transitions based solely on MS/MS spectral behavior.

To assess the performance of the METLIN-based empirical approach, we compared calculated MRM transitions against experimentally optimized transitions across a diverse set of metabolites (Table 1). These include amino acids in positive ion mode, TCA intermediates in negative ion mode, and other biologically relevant compounds detected in both modes. For each metabolite, quantifier and qualifier ions were selected using either the METLIN-intensity-based algorithm or traditional instrument-specific optimization. The table highlights the selected fragment ions and their corresponding optimal collision energies (CEs), enabling direct comparison between predicted and experimental transitions.

**Table 1:**
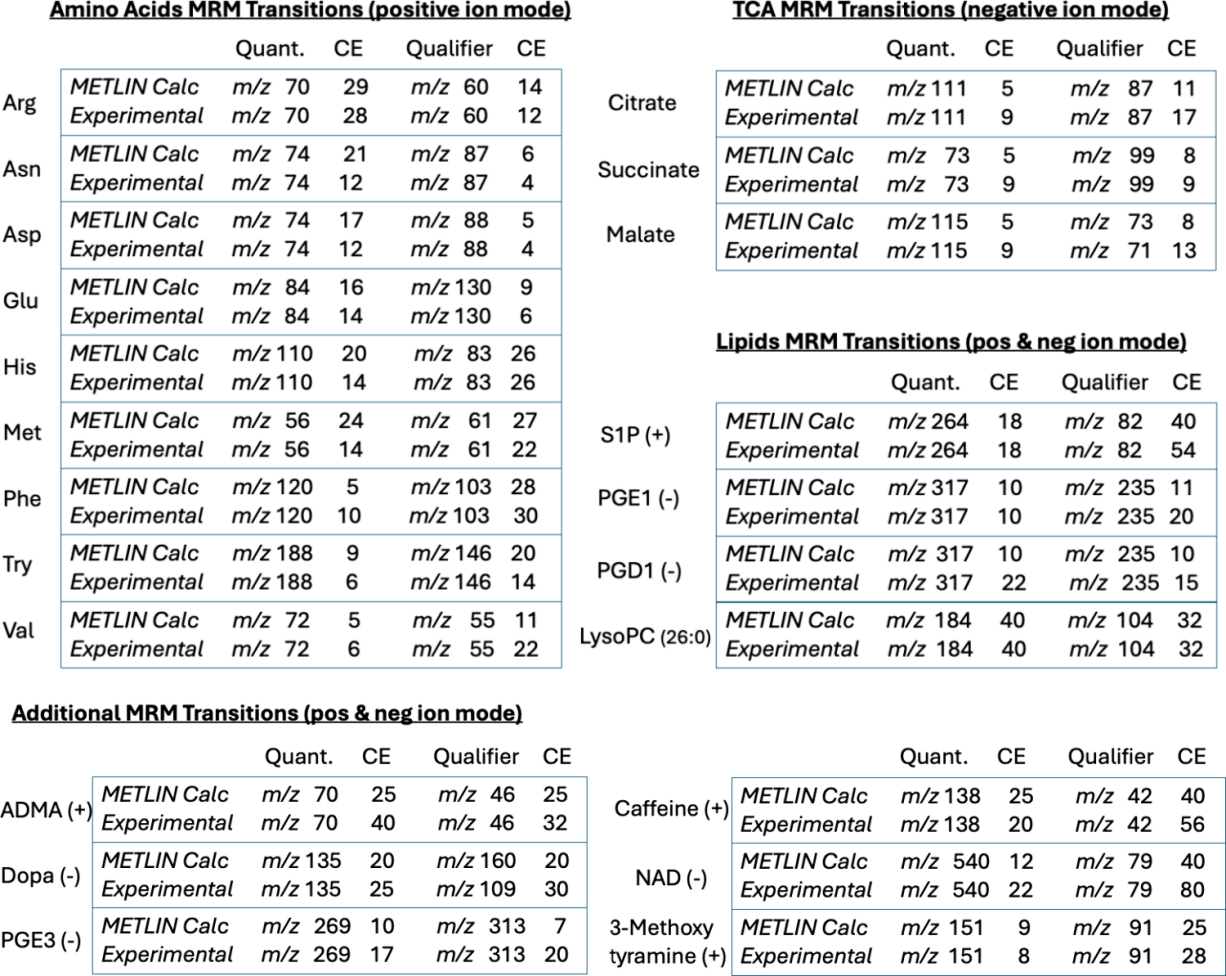
Comparison of METLIN-Calculated and Experimental MRM Transitions for Selected Molecules in Positive and Negative Ion Modes^a^

a Multiple reaction monitoring (MRM) transitions were generated using METLIN’s absolute intensity MS/MS data and compared with experimentally optimized transitions for amino acids (positive ion mode), tricarboxylic acid (TCA) cycle intermediates (negative ion mode), and other common lipids and molecules in both positive and negative ion modes. For each compound, the quantifier and qualifier ions (m/z) are shown along with their respective optimal collision energies (CE). METLIN-calculated values were derived using empirical CE-intensity profiles, while experimental values were determined independently with triple quadrupole mass spectrometers of different manufacturer and model types. The chosen malate qualifier ions (m/z 71 and 73) chosen in the experimental and METLIN calculated separately, both ions were observed in the METLIN MS/MS data, and the latter (m/z 73) was chosen.

The agreement between METLIN-calculated and experimental transitions was consistently high. In all but one case (malate qualifier was chosen at 73 fragment *m*/*z* rather than 71 *m*/*z*), the same fragment ions were selected or fell within a small *m*/*z* tolerance. Furthermore, the empirically determined CEs closely matched or approximated the experimentally optimized CEs, often differing by only a few electron volts. Notably, even for complex molecules such as NAD, the METLIN-based pipeline identified the same transitions as those optimized by expert users. These findings underscore the utility of leveraging large-scale empirical MS/MS data to streamline MRM assay development while maintaining alignment with traditional experimental standards.

The METLIN 960K MRM pipeline demonstrates high fidelity in replicating experimentally optimized transitions where it matched all the quantifier masses, and over 90% of both qualifier masses and CE values. Importantly, even discrepancies (like for malate or NAD) are scientifically reasonable and often reflect differences in optimization goals (intensity vs selectivity) or instrument configuration. This analysis supports the robustness and practical utility of the AI-optimized METLIN MRM library for transition selection, even in cases lacking commercial standards or experimental CE data. Ongoing large-scale validation efforts are expected to further benchmark and refine the METLIN 960K MRM resource, with results to be presented in a future publication.

To quantitatively evaluate the predictive accuracy of the METLIN 960K MRM pipeline, we compared the algorithmically derived transitions to experimentally optimized MRM data across a diverse set of compounds (Table 1). Overall, the performance across key metrics: agreement of quantifier and qualifier *m*/*z* values, predicted versus experimental collision energy (CE), and full transition set overlap. The METLIN pipeline correctly predicted the quantifier ions and most of the qualifier ions and CE values typically within 7 eV, with the qualifier (less intense ion) prediction being less accurate. Although it should be noted that discrepancies often reflect alternate optimization priorities (e.g., selectivity vs intensity) or differences in instrumentation. In one case (LysoPE 13:0) the quantifier and qualifier ions were flipped. Larger-scale validation is ongoing to evaluate accuracy and to further refine the algorithm. While METLIN MRM predicts empirically validated transitions that perform robustly across platforms, instrument-specific optimization may yield alternative transitions with comparable performance. In such cases, both the METLIN-derived and instrument-optimized transitions are likely to be valid, differing mainly in their relative emphasis on sensitivity, selectivity, or dwell-time constraints.

While agreement between METLIN-predicted and experimental transitions was consistently high, several limitations should be noted. First, although the algorithm can predict transitions using METLIN’s MS/MS data, absolute quantification still requires calibration with authentic compounds. Second, discrepancies in qualifier ion selection or CE values (e.g., malate, NAD) reflect the fact that optimization priorities can differ across instruments and users, particularly when balancing intensity versus selectivity. Finally, as retention times are not part of the METLIN 960K MRM resource, the development of dynamic MRM workflows for optimal sensitivity will necessitate independent determination of retention times.

To highlight the breadth and novelty of the METLIN 960K MRM library,^15,16^ Figure 4 illustrates its scale, composition, and accessibility. Panel 4a shows that the 2025 METLIN MRM resource (large blue sphere) far exceeds the size of earlier libraries, including the 2018 METLIN-MRM and other publicly available repositories,^3–10^ with only limited overlap in compound coverage. **Panel 4b** emphasizes that most compounds in METLIN 960K are not commercially available as single-sourced standards; many were obtained through bulk acquisitions, collaborations, or custom synthesis, underscoring the empirical investment underlying this resource. **Panel 4c** places the library in context against other repositories, revealing that METLIN 960K—with nearly one million small molecules—dwarfs proteomic-focused collections such as SRM Atlas and MRMaid, as well as previous small-molecule MRM databases. **Panel 4d** highlights the chemical breadth of the library, spanning more than 350 distinct classes and offering broad representation across the molecular landscape. **Panel 4e** underscores the diversity of sources, with >1.2 million standards sourced across organizations including academic consortia, government agencies, biotech companies, instrument vendors, pharmaceutical firms, agricultural organizations, among others.

**Figure 4:**
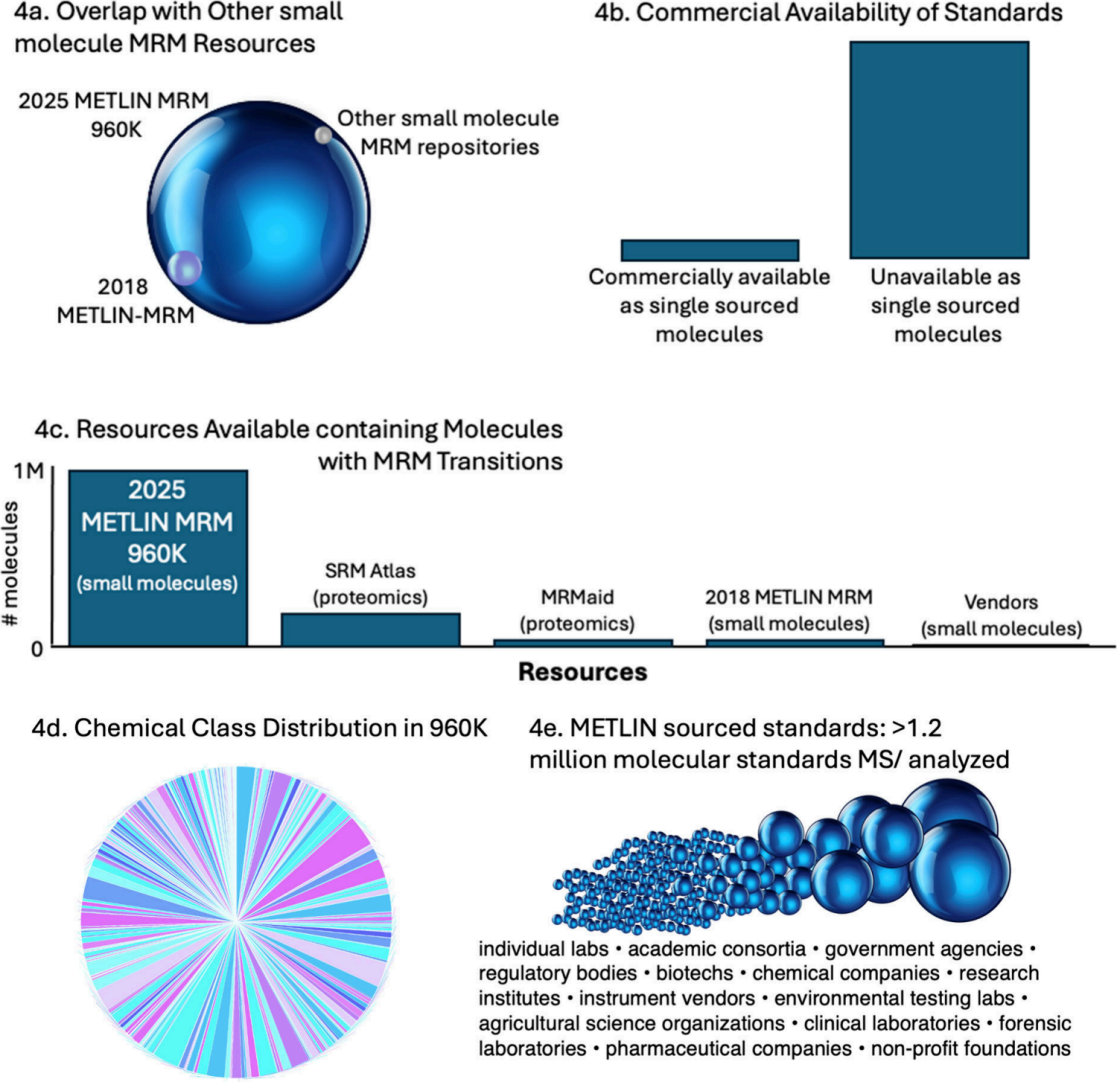
Scope and novelty of the METLIN MRM 960K library. (a) Overlap of the 2025 METLIN MRM 960K library (large sphere) with previous MRM resources, including the 2018 METLIN-MRM library (small blue sphere) and other publicly available MRM repositories (small gray sphere). The size of each sphere represents relative database scale. (b) Commercial standard availability for the METLIN MRM 960K library compounds. The commercial availability of the molecules as single sourced molecules is limited (left bar).The majority of molecules are available as commercial standards only when acquired in large sets (right bar), while others are not commercially available. (c) Current known MRM resources with the number of molecules containing MRM transitions. (d) Chemical class distribution using Classyfire of the METLIN library covering over 350 chemical classes (20), these include fatty acyls, steroids and steroid derivatives, carboxylic acids and derivatives, organonitrogen compounds, prenol lipids, organooxygen compounds, glycerophospholipids, glycerolipids, sphingolipids, organoheterocyclic compounds, benzene and substituted derivatives, phenols and derivatives, indoles and derivatives, pyridines and derivatives, alkaloids and derivatives, hydrocarbons, organic acids and derivatives, amino acids, peptides, and analogues, nucleosides, nucleotides, and analogues, carbohydrates and carbohydrate conjugates, flavonoids and polyphenols, quinones and hydroquinones, phenylpropanoids and polyketides, organohalogen compounds, organosulfur compounds, organophosphorus compounds, organosilicon compounds, lignans, neolignans, and related compounds, polyketides and macrolides, fatty acid esters, eicosanoids, terpenoids, coumarins and derivatives, acylcarnitines, hydroxy acids and derivatives, aromatic heteropolycyclic compounds, and heteroaromatic compounds. (e) Broad distribution of standards sourced from a multitude of organizations as represented by the spheres.

Chemical diversity analysis performed using ClassyFire^18^ ontology identified more than 350 distinct chemical classes represented within the METLIN 960K MRM library, spanning metabolites, lipids, xenobiotics, natural products, drugs, and synthetic intermediates (**Panel 4d**). To place this in context, community databases such as HMDB^19^ and LIPID MAPS^20^ provide comprehensive structural and biological annotations and were used here to classify molecular types represented in METLIN. However, as these resources do not contain validated MRM or collision-energy data, they complement rather than overlap with METLIN’s empirical MRM framework. Benchmarking of transition performance was therefore performed against SRM Atlas, MRMaid, and vendor-supplied MRM libraries, which represent the current empirical standards for small-molecule MRM data.

Together, these panels demonstrate that METLIN 960K is not only the largest small-molecule MRM library available but also the most chemically and institutionally diverse. Unlike existing resources such as SRMAtlas and MRMaid, which primarily target peptides, METLIN 960K uniquely provides more than 3 million empirically derived small-molecule transitions in both positive and negative ionization modes. Commercial libraries typically contain fewer than 5,000 small molecules and are often restricted to proprietary kits with limited transparency and scope. In contrast, the METLIN 960K library is openly accessible, broadly applicable, and standardized, offering a scalable platform for quantitative mass spectrometry. Its integration of empirical MS/MS data with AI-guided optimization expands the landscape of MRM transition availability, enabling sensitive detection of both common and rare molecular targets.

To demonstrate the practical utility of METLIN-predicted transitions for biologically relevant compounds, we analyzed β-*N*-methylamino-l-alanine (BMAA),^21^ a neurotoxic nonproteinogenic amino acid implicated in neurodegenerative disease. Figure 5 presents the predicted transitions (quantifier: *m*/*z* 44.0; qualifier: *m*/*z* 102.1) and their associated collision energies, which closely align with experimentally validated values. The CE-dependent MS/MS spectra further highlight the distinct fragmentation behavior of BMAA across collision energies, underscoring the accuracy of the spline-based prediction framework. The ability to generate high-confidence MRM transitions for rare and costly compounds such as BMAA illustrates the value of METLIN’s comprehensive spectral database in enabling precision quantitation even for molecules absent from standard libraries.

**Figure 5:**
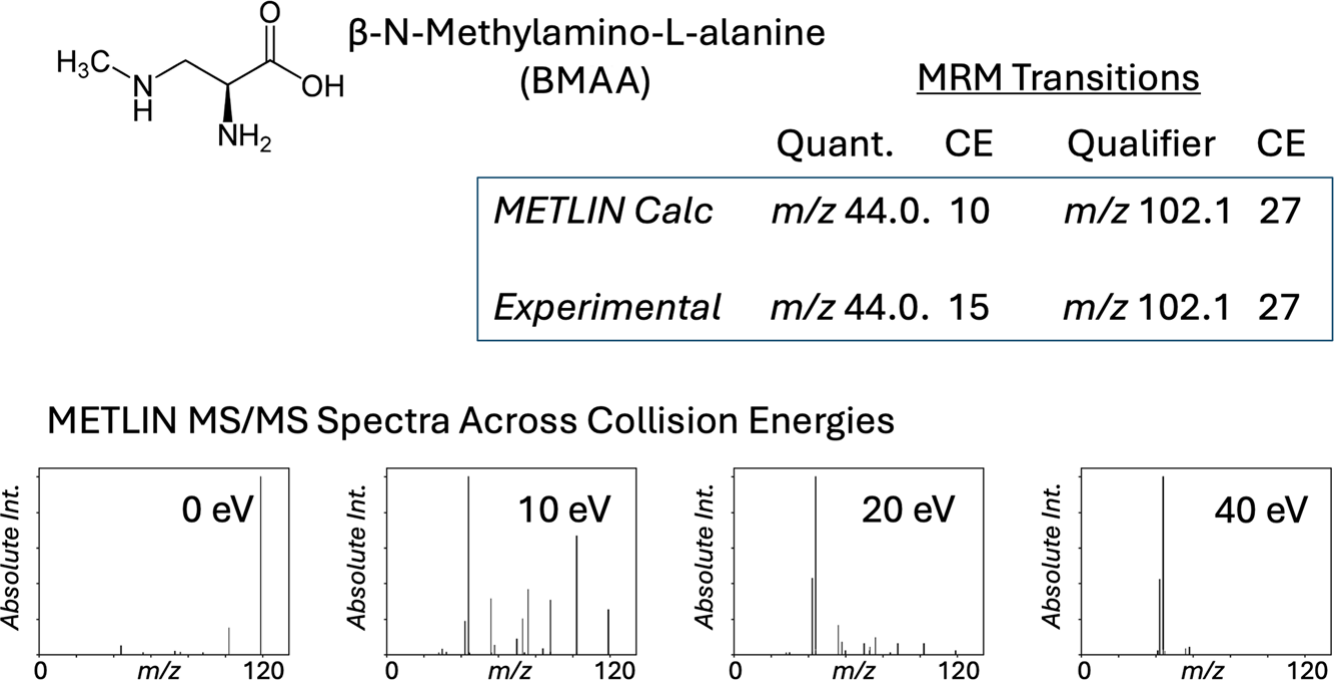
METLIN-guided MRM transition prediction and validation for β-*N*-methylamino-l-alanine (BMAA). Predicted and experimentally optimized MRM transitions for BMAA are shown, including quantifier (*m*/*z* 44.0) and qualifier (*m*/*z* 102.1) ions with their corresponding collision energies (CEs). While the predicted and experimental qualifier transitions match well, the predicted CE for the quantifier ion was slightly lower (10 eV vs 15 eV). Bottom: METLIN MS/MS spectra for BMAA (MID 435578) across four collision energies (0, 10, 20, and 40 eV), demonstrating energy-dependent fragmentation patterns. These results illustrate the ability of the METLIN 960K MRM pipeline to replicate experimental transitions using only intensity modeling.

From a practical standpoint, MRM optimization—whether via software or manual tuning—often requires repeated injections and substantial quantities of analyte. For high-value or scarce compounds, this can make experimental MRM generation impractical, positioning METLIN’s predictive approach as a cost- and resource-efficient alternative. Moreover, time and cost demand scale rapidly as panel size increases; developing a 10–20 compound panel through traditional methods can require thousands of dollars’ worth of material, even for compounds priced at $50–$100 per mg. For pilot studies, such investment may be difficult to justify, making METLIN’s ready-to-use MRMs a substantial time and cost saver.

More broadly, METLIN MRM should be viewed as an enabling platform rather than a replacement for traditional MRM development. Its primary strength lies in accelerating pilot studies and discovery-driven experiments by providing empirically derived transitions that can be deployed rapidly at minimal cost and with no requirement for authentic standards. Conventional optimization using standards remains essential for final assay qualification and absolute quantitation, but METLIN MRM bridges the gap between discovery and targeted validation, dramatically shortening the path from metabolite observation to quantitative measurement.

To further validate the scalability of METLIN-predicted transitions, we applied the framework to a mixture of more than 80 authentic standards spanning diverse chemical classes.^17^ Notably, we had not previously optimized MRM transitions for these compounds, yet METLIN-derived quantifier and qualifier ions with associated CEs enabled robust chromatographic detection across the entire panel. As shown in Figure 6, the compounds were detected at both 1 μM and 1 nM concentrations. Reliable detection down to the low nanomolar range underscores the sensitivity of the approach and demonstrates that large, targeted panels can be rapidly assembled without the need for compound-specific optimization. This highlights the practical utility of the METLIN 960K MRM resource in enabling scalable, high-throughput targeted analyses where standards are limited or unavailable.

**Figure 6:**
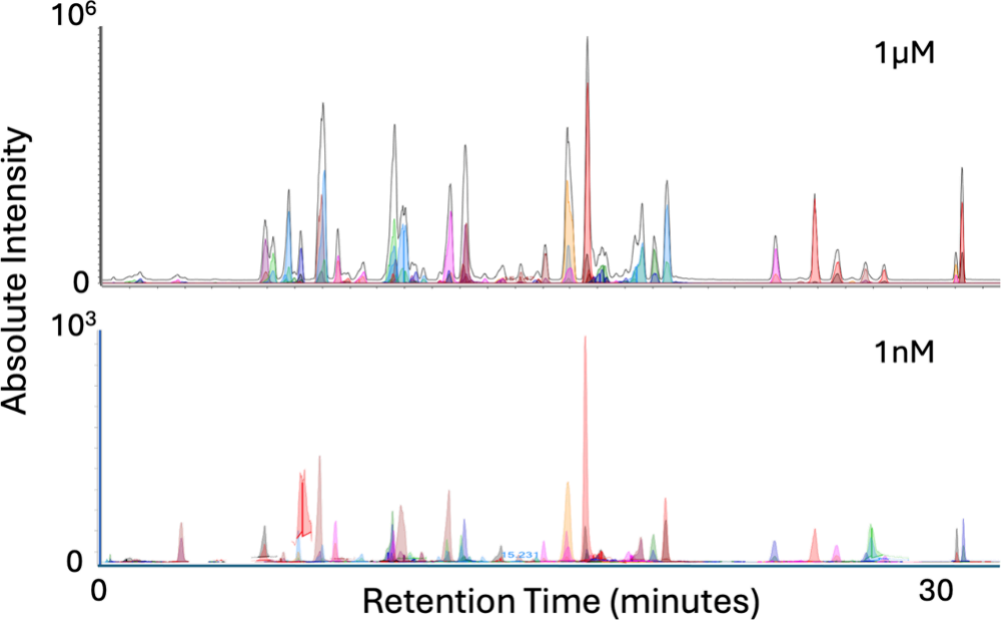
Application of METLIN-derived MRM transitions for targeted analysis of a mixture of more than 80 authentic standards without individual QqQ compound-specific optimization. Extracted ion chromatograms are shown for all transitions at 1 μM (top) and 1 nM (bottom). Despite the absence of optimized MRM methods, METLIN-predicted quantifier and qualifier transitions enabled robust detection and chromatographic separation of all compounds across both concentration levels. Reliable detection down to 1 nM underscores the sensitivity and practical utility of the METLIN MRM resource. The analyzed standards represent a chemically diverse panel, including sphingosine-1-phosphate, d-erythro-sphinganine, probenecid, chloroquine, cimetidine, oleamide, amoxicillin, tetracycline, caffeine, camosine, *N*-pentadecylamine, sulpiride, biotin, glu-leu, dihydroceramide, palmitoyl dopamine, oleoyl dopamine, anandamide, arachidonoylglycine, aminoantipyrine, octadecanamine, spermidine, quinacrine, theophylline, raclopride, *N*,*N*-dimethylsphingosine, decanoylcarnitine, arachidonamide, aminophenanthridine, fluensulfone, heclin, sphingosine, *N*-oleoylethanolamine, takinib, fenbendazole sulfone, cipargamin, pirlimycin, epioxytetracycline, C8 ceramide, *N*-oleoyl glycine, fraxinol, rolipram, inauhzin, ryuvidine, indolmycin, nicaraven, nexturastat A, tubacin, and erythromycin A oxime, among others.

This experiment also addresses the breadth of chemical validation: while the initial proof-of-concept used a smaller set of compounds, the expanded analysis confirms that METLIN MRM transitions generalize across a larger and chemically diverse panel. These results strengthen the evidence that the METLIN 960K MRM resource is broadly applicable, enabling scalable, high-throughput targeted analyses where standards are limited or unavailable.

These findings confirm that METLIN 960K transitions can be applied across diverse chemical classes and concentration ranges. However, several practical limitations remain. The database does not capture matrix effects, which can alter transition performance in complex biological samples. Retention times are not provided, requiring users to establish them experimentally if dynamic MRM approaches are to be employed. Additionally, while the current validation spans over 100 compounds, large-scale benchmarking across thousands of molecules will be needed to quantitatively determine performance across the chemical space.

## Future Work

The METLIN 960K MRM pipeline demonstrates strong agreement with experimentally optimized transitions in the current benchmarking set, which includes a representative range of chemical classes, ionization modes, and mass ranges, providing proof-of-concept. Further validation is provided in Figure 6 where targeted analyses at low sensitivity was achieved with the METLIN MRMs. However, given the unprecedented scale of the MRM library (MRM transitions in both positive and negative ionization modes of >960,000 compounds), broad-scale validation will be essential to fully determine quantitatively the performance across the chemical space. This expanded benchmarking is already in progress. These studies will also enable refinement of fragment selection criteria and collision energy prediction models for optimal performance. It is important to note that no adduct designation is included in this resource; transitions are reported solely by precursor *m*/*z* and corresponding fragment ions. While this does not affect the accuracy of precursor–fragment pairing, users requiring adduct-specific information can readily calculate it from the molecular formula.

## Conclusion

The METLIN 960K MRM resource transforms targeted mass spectrometry by delivering empirically derived, AI-refined transitions for approximately 960,000 compounds based solely on experimental MS/MS data from authentic standards. By eliminating reliance on standard-based transition optimization,^22^ it provides high-confidence quantifier/qualifier ions and optimized collision energies, including for rare, costly, or commercially unavailable molecules. Leveraging high-resolution MS/MS spectra acquired across multiple collision energies, the framework achieves strong concordance with experimentally validated transitions while enabling scalable, vendor-independent MRM assay design.

Validation on exemplars such as BMAA, > 30 individual molecules, and mixtures of >80 diverse standards down to 1 nM confirms that the predictive framework produces robust MRMs. Importantly, METLIN MRM is not intended to supplant traditional targeted MRM workflows that rely on authentic standards and calibration curves for full quantitative validation. Rather, it serves as a complementary resource that accelerates pilot-study design and early stage discovery by providing empirically grounded transitions that can be implemented immediately at low cost in both research and preclinical contexts. This approach also holds immediate applicability for preclinical drug development studies, where authentic metabolite and impurity standards are often unavailable. In this way, METLIN MRM expands access to quantitative assays while preserving the rigor of conventional validation when absolute quantification is required.

Transitions are reported in a precursor *m*/*z*–centric format without explicit adduct designation. This choice minimizes coding errors and ambiguity while remaining compatible with complementary resources and QqQ software that can accept transitions without specified adducts.

The analytical and modeling components were developed within the AI BioSync framework, an XCMS enhancement that provides a collective environment of AI and machine-learning tools designed to decipher spectral data for biological and analytical relevance. By integrating empirical MS/MS data with spline modeling refined through AI BioSync, METLIN 960K MRM enables targeted assay design for an unprecedented molecular scope. The resulting data sets are compact, fast to process, and inherently stable over time, facilitating cross-study and cross-platform use. Ongoing benchmarking will extend validation and refine transition and CE models. Together, these advances establish METLIN 960K MRM as a practical, accurate, and future-proof platform for quantitative analysis across research, clinical, and applied settings.

Key advantages:

- Empirically derived, AI-guided MRM transitions for 960K compounds.
- Optimized MRM transitions are provided without the need to acquire standards, enabling rapid targeted assay development for rare, costly, or unavailable molecules.
- Validation across diverse standards with sensitivity to 1 nM.
- Precursor *m*/*z*–centric reporting eliminates adduct-related ambiguity while maintaining compatibility with standard QqQ and MRM software workflows.
- Vendor-independent platform enabling robust, scalable quantitative analysis.
